# Comparative study on the microleakage performance of different pit and fissure sealants

**DOI:** 10.6026/973206300220744

**Published:** 2026-02-28

**Authors:** Shivani Mishra, Shivani Singh, Kanchan Sharma, Sheetal Mohan Jadhav, Soundarya Vishwanathan, Ratheesh M.S, Pratik Surana

**Affiliations:** 1 Department of Dentistry, Virendra Kumar Sakhlecha Government Medical College, Neemuch, Madhya Pradesh, India; 2 Consultant Orthodontist, Superior Smile Dental Clinic, Kanke Road, Ranchi, Jharkhand, India; 3 Department of Orthodontics and Dentofacial Orthopaedics, Awadh Dental College and Hospital, Jamshedpur, Jharkhand, India; 4 Department of Prosthodontics and Crown and Bridge, Bharati Vidyapeeth (Deemed to be University) Dental College and Hospital, Navi Mumbai, Maharashtra, India; 5 Department of Pediatric and Dentistry, The Oxford Dental College, Bangalore, Karnataka, India; 6 Department of Pediatric and Preventive Dentistry, MES Dental College, Perinthalmanna, Kerala, India; 7 Department of Pedodontics and Preventive Dentistry, Maitri College of Dentistry and Research Centre, Durg, Chhattisgarh, India

**Keywords:** Microleakage, pit and fissure sealants, nano silver reinforced sealant

## Abstract

The variable sealing ability of currently available pit and fissure sealants remains a major challenge in preventing occlusal caries in susceptible teeth. Microleakage at the tooth-sealant interface compromises retention and caries-preventive effectiveness, thereby affecting long-term clinical success. Therefore, it was of interest to compare microleakage in three pit and fissure sealants using 30 extracted permanent premolars divided into resin-based, glass ionomer and nano-silver-reinforced groups (n = 10). Following application, thermocycling and dye immersion, samples were evaluated under a stereomicroscope. Glass ionomer showed the highest microleakage, whereas the nano-silver-reinforced sealant showed the least.

## Background:

Pit and fissure caries is a microbial disease caused by changes in bacterial biofilm when exposed to fermentable carbohydrates, disrupting the balance between demineralization and remineralization [1, 2]. Deep pits and fissures on occlusal surfaces are particularly susceptible to dental caries, accounting for 56-70% of caries in children aged 5-17 [3]. According to a panel of experts from the American Dental Association and American Academy of Pediatric Dentistry, pit and fissure sealants are more effective than fluoride varnish and the absence of sealants in preventing and managing occlusal caries in children and adolescents [4]. Modern dentistry emphasizes preventive treatments like fluoride, which primarily protect smooth surfaces rather than occlusal ones, where structural defects encourage plaque retention and reduce fluoride effectiveness [5]. To address this, pit and fissure sealants have been developed as an effective, minimally invasive preventive measure. These sealants isolate pits and fissures from bacteria, create a mechanical barrier and prevent plaque accumulation. Applying sealants significantly reduces caries risk compared to non-sealed teeth and is more cost-effective than restorative treatments [6, 7]. Resin-based materials are commonly used as sealants, including various filled and unfilled types, some of which release fluoride.

The primary component is bisphenol A-glycidyl methacrylate (Bis-GMA) resin, typically applied with 37% phosphoric acid for etching. When moisture is properly controlled, these sealants demonstrate effective sealing due to their low viscosity, allowing deep penetration into pits and fissures and creating a resin-impregnated enamel layer. Although the effectiveness of sealants has been widely researched, no individual material is deemed ideal. Glass-based sealants are often recommended for sealing pits and fissures because they are less affected by moisture [8, 9]. Recently, silver nanoparticles have been introduced into sealants. The incorporation of these nanoparticles in pit and fissure sealants may provide an alternative means to inhibit the caries formation process, a widespread issue. However, there is ongoing debate about how the addition of silver nanoparticles impacts the physical properties of the sealants [10]. Microleakage poses a significant challenge for restorative materials and can result in issues such as secondary cavities, damage to the dental pulp, sensitivity after treatment, color changes at the edges and restoration fractures [11]. Ganesh and Shobha observed that how well a sealant fits with the enamel are a key factor in its effectiveness and longevity. Any compromise in the sealant's edge can allow bacteria to accumulate beneath these repaired fissures, potentially leading to decay forming under the restoration. Therefore, the effectiveness of pit and fissure sealants largely relies on their ability to stay attached and bond properly to the enamel surfaces [12]. Therefore, it is of interest to assess and compare microleakage in three different pit and fissure sealants: Resin-Based Sealant, Glass Ionomer Sealant and Nano Silver Reinforced Sealant.

## Materials and Methods:

Present *in-vitro* study involved 30 freshly extracted premolars that were removed for orthodontic reasons. Only teeth with undamaged biting surfaces, free from decay, repairs and cracks, were included. Teeth with developmental issues were not part of the study. Before testing, all teeth were cleaned and stored in distilled water at room temperature. The specimens were then randomly divided into three equal groups: Group I consisted of teeth to be treated with a traditional pit and fissure sealant (Helioseal F; Ivoclar Vivadent AG, Liechtenstein), Group II a glass ionomer-based sealant (Fuji VII; GC Corporation, Japan) and Group III was for teeth to be sealed with a Nano Silver Reinforced Sealant (Kids-e-Pit and Fissure Sealant, Kids-e-dental, Mumbai, India).

## Preparation of samples:

The occlusal surfaces of samples in Groups I and III were treated with 37% phosphoric acid for 30 seconds. Following the etching, the surfaces were rinsed and allowed to air dry. Before applying the sealant, a bonding agent (Single Bond 2, 3M, USA) was applied to the fissures with a microbrush and cured for 10 seconds, as per the manufacturer's instructions. The sealant was then applied to the fissures according to the manufacturer's guidelines. A periodontal probe was gently moved along the fissures to ensure there were no voids or trapped air. Finally, a light cure unit was used to polymerize the occlusal surfaces. In Group II (GC Fuji VII), the occlusal surfaces were conditioned for 20 seconds, rinsed and dried by blotting. GC Fuji VII was mixed per manufacturer instructions, applied with a plastic instrument and spread into the pits and fissures using a fine brush, allowing 10 seconds for flow. The sealant was then light cured for 20 seconds and petroleum jelly was applied to the surface.

## Microleakage testing:

After sealant placement, the samples were stored in artificial saliva for 24 hours at room temperature. They were then thermocycled for 250 cycles between 5°C and 55°C. Sticky wax was applied to each apex and the teeth were coated with two layers of nail varnish, leaving a 2 mm margin around the sealant. The specimens were immersed in 5% methylene blue for 24 hours, rinsed to remove excess dye and sectioned buccolingually using a high-speed handpiece with a diamond disk under water spray. The sections were examined under a stereomicroscope at 10x magnification and images were recorded. The examination process utilized a 4-point scoring system conducted by a single observer, following the Ovrebo and Raadal criteria for evaluating dye penetration [13].

## The criteria for grading microleakage are as follows:

[1] Score 0: No dye penetration

[2] Score 1: Dye penetration limited to the outer half of the enamel-sealant interface

[3] Score 2: Dye penetration occurring in the inner half of the enamel-sealant interface

[4] Score 3: Dye penetration extending into the underlying fissure.

Result was collected, organized into tables and analyzed statistically using SPSS 24 software.

## Results and Discussion:

The present study evaluated and compared the microleakage of three different pit and fissure sealants-resin-based, glass ionomer-based and nano-silver reinforced-using a dye penetration method. The distribution of microleakage scores is presented in Table 1, which demonstrated clear differences among the three groups. The overall chi-square analysis revealed a statistically significant difference in microleakage scores among the sealant materials (χ^2^ = 16.47, Df = 6, p < 0.05), indicating that the type of sealant material significantly influenced marginal sealing ability. Group II (glass ionomer-based sealant) showed the highest degree of microleakage, with most samples scoring 2 or 3, indicating extensive dye penetration along the sealant-enamel interface. In contrast, Group I (resin-based sealant) exhibited moderate microleakage, with most specimens scoring 0 or 1. Group III (nano-silver reinforced sealant) demonstrated the least microleakage, with the majority of samples showing minimal or no dye penetration. Pairwise intergroup comparisons Table 2 further supported these findings. Microleakage in Group II was significantly higher than in Group I (p = 0.044) and Group III (p = 0.021). However, no statistically significant difference was observed between Group I and Group III (p = 0.950), suggesting comparable sealing performance between resin-based and nano-silver reinforced sealants; although the latter showed a more favorable distribution of lower leakage scores. For many years, the occlusal surfaces-especially the pits and fissures of posterior teeth-have been identified as highly prone to dental caries. This heightened vulnerability is largely due to their complex morphology. Although pits and fissures differ in shape, they are typically very narrow (around 0.1 mm wide) and winding, making them ideal sites for the accumulation of food debris and bacteria. Because of their structure, they are difficult to clean mechanically; in fact, a typical toothbrush bristle (0.2 mm thick) is too large to effectively enter most fissures [14, 15]. Additional factors also contribute to the increased caries risk in these areas, including limited access for saliva, the close proximity of fissure bases to the dentino-enamel junction and the presence of pellicle and debris within fissures. To counteract this high susceptibility, pit and fissure sealants were introduced [16]. Sealants help prevent caries primarily by physically sealing these grooves, blocking bacterial colonization and restricting fermentable carbohydrates from reaching any bacteria that may remain. Numerous studies have demonstrated a strong association between the use of sealants and reduced caries incidence [15, 17]. Various local factors can affect how well sealants penetrate pits and fissures, regardless of the material used. Potential contaminants on the tooth surface-including the salivary pellicle, carbohydrate-metabolism by-products and lubricating oil from the airotor handpiece-have been identified as contributors to reduced penetration. These contaminants can occlude the natural porosity of enamel, preventing proper penetration and adhesion of the sealant. Therefore, prophylaxis (cleaning) prior to sealant application is essential to remove these barriers and enhance sealant adaptation, which is also important to minimize marginal leakage. In our study, we selected maxillary and mandibular premolars extracted for orthodontic reasons; all teeth were caries-free and free from developmental defects, enamel microfractures, or discoloration [18]. For storage of the extracted teeth, we used artificial saliva - a solution that preserves the tooth without altering protein structure or enamel morphology- to ensure that the enamel remained unaltered prior to sealant application. The current investigation compared the microleakage of three pit and fissure sealants-Resin-Based, Glass Ionomer and Nano-Silver Reinforced. The dye-penetration method has frequently been used to assess marginal leakage at the sealant- enamel interface [19, 20]. In the present study, we used a qualitative dye-penetration technique. The infiltration of dye serves as an indicator of imperfect sealing: dye appearing in the under- penetrated zones of etched enamel suggests a potential microleakage pathway [21]. Clinically, this may imply that any etched area left exposed- especially if the sealant is partially or completely lost - could favor development of caries by allowing microleakage under the sealant. The findings of this study demonstrated that the Nano Silver reinforced sealant exhibited the least microleakage, followed by the resin-based, while the glass ionomer sealant showed greatest degree of dye penetration.

The superior performance of the Nano Silver reinforced sealant may be attributed to its modified resin matrix and the presence of nano-sized silver particles, which enhance both physical and antimicrobial properties. Nano-silver incorporation is known to improve the mechanical integrity of resin materials by reducing polymerization shrinkage and increasing filler density. These factors collectively enhance marginal adaptation and reduce the formation of interfacial gaps. Additionally, the antimicrobial activity of silver nanoparticles may reduce bacterial colonization at the sealant margins, potentially contributing to improved interfacial stability over time. Although antimicrobial activity does not directly influence microleakage in an *in vitro* dye penetration model, improved polymer network formation and better wetting characteristics likely contribute to the reduced leakage observed [22]. The resin-based sealant (Helioseal F) demonstrated moderate microleakage, which aligns with findings from previous studies that have shown resin sealants to generally perform well due to their micromechanical retention via acid etching and strong bond strength to enamel. However, polymerization shrinkage inherent to resin materials may still lead to marginal gaps despite optimal etching and curing procedures. Factors such as operator technique, the viscosity of the sealant and the degree of conversion during polymerization may also influence the microleakage results. Fluoride content in Helioseal F may enhance caries prevention but does not directly improve sealing ability, which could explain its intermediate performance status between the nano-modified resin and GIC based Sealants. In contrast, Fuji VII exhibited the highest microleakage among the three groups. Glass ionomer materials are known for their chemical adhesion to tooth structure and their sustained fluoride release, both of which offer clinical advantages, particularly in situations where moisture control is challenging. However, their lower mechanical strength, higher solubility and sensitivity to early moisture contamination may compromise marginal integrity. *in vitro*, where thermocycling simulates thermal expansion and contraction stresses, glass ionomers may exhibit increased crack formation or marginal breakdown. The use of conventional GIC rather than resin-modified GIC may also have contributed to increased leakage due to weaker initial setting characteristics and vulnerability to hydration and dehydration during testing [23]. Overall, the findings reinforce the importance of material selection in preventive dentistry. While glass ionomer sealants offer advantages such as fluoride release and tolerance to moist conditions, their susceptibility to microleakage may limit their long-term sealing ability [24]. Resin-based sealants remain a dependable option in situations where optimal isolation can be achieved [25]. The Nano Silver reinforced sealant, demonstrating the least microleakage in this study, appears to offer promising improvements in marginal adaptation, potentially due to enhancements in physical properties obtained through nanotechnology [26]. These findings suggest that nano-modified materials may represent a valuable advancement in sealant technology, though further long-term clinical research is required to confirm whether the *in vitro* benefits translate into superior *in vivo* outcomes. Future research should aim to correlate *in vitro* microleakage findings with *in vivo* outcomes, as current evidence shows a poor relationship between the two. While *in vitro* studies are useful for comparing the sealing ability of materials, true assessment of microleakage requires clinical trial to evaluate micromechanical bond and long-term performance of fissure sealants under real oral conditions. Joshi *et al.* also emphasized the importance of correlating laboratory-based microleakage results with clinical outcomes [27].

## Conclusion:

The Nano-Silver reinforced sealant showed the least microleakage among the three materials tested, indicating superior marginal adaptation and sealing ability. These findings suggest advantages of nano-enhanced sealants, although clinical investigations are required to verify long-term performance.

## Figures and Tables

**Table 1:** Distribution Microleakage score of three groups

**Score**	**Groups**			**χ^2^**	**Df**	**P**
	**I**	**II**	**III**			
Score 0	4 (40%)	0 (0%)	5 (50%)	16.47	6	<0.05*
Score 1	4 (40%)	2 (20%)	4 (40%)			
Score 2	2 (20%)	3 (30%)	1 (10%)			
Score 3	0 (0%)	5 (40 %)	0 (0%)			
* Significant

**Table 2:** Inter-Group (Pairwise) Comparison of Microleakage

**Comparison**	**χ^2^ Value**	**df**	**p-value**	**Significance**
I vs II	8.1	3	0.044*	Significant
I vs III	0.35	3	0.95	Not Significant
II vs III	9.7	3	0.021*	Significant
